# An Imaging Approach to Identify Mechanisms of Resistance to Pineapple Fruitlet Core Rot

**DOI:** 10.3389/fpls.2019.01065

**Published:** 2019-09-10

**Authors:** Bastien Barral, Marc Chillet, Mathieu Léchaudel, Marc Lartaud, Jean-Luc Verdeil, Geneviève Conéjéro, Sabine Schorr-Galindo

**Affiliations:** ^1^CIRAD, UMR Qualisud, Saint-Pierre, France; ^2^Qualisud, Univ Montpellier, CIRAD, Montpellier SupAgro, Univ d’Avignon, Univ de La Réunion, Montpellier, France; ^3^CIRAD, UMR Qualisud, Capesterre-Belle-Eau, France; ^4^CIRAD, UMR AGAP, Montpellier, France; ^5^PHIV, BPMP, Univ Montpellier, INRA, CNRS, SupAgro, Montpellier, France

**Keywords:** fruitlet core rot, disease resistance, carpel margin, septal nectaries, ferulic acid, coumaric acid, *Ananas comosus*

## Abstract

Fruitlet core rot is one of the major postharvest disease of pineapple (*Ananas comosus* var. *comosus*). In the past, control strategies were designed to eliminate symptoms without addressing their causes or mechanisms, thus achieving only moderate success. In this study, (i) we focused on the anatomy of the fruitlets in the resistant “MD-2” and susceptible “Queen” pineapple cultivars; (ii) we identified the key role of the carpel margin in the infection process; (iii) we identified the key role of the sinuous layer of thick-walled cells in the inhibition of *Fusarium ananatum* colonization; and (iv) we linked the anatomy of the fruitlets with the phenolic content of cell walls. The fruitlet anatomy of the two cultivars was studied using X-ray, fluorescence, and multiphoton microscopy. Sepals and bracts were not perfectly fused with each other, allowing the pathogen to penetrate the fruit even after flowering. In fact, the fungi were found in the blossom cups of both cultivars but only became pathogenic in the flesh of the “Queen” pineapple fruit under natural conditions. The outer layer of the “MD-2” cavity was continuous with thick cell walls composed of ferulic and coumaric acids. The cell walls of the “Queen” blossom cup were less lignified at the extremities, and the outer layer was interspersed with cracks. The carpel margins were fused broadly in the “MD-2” pineapple, in contrast to the “Queen” pineapple. This blemish allows the fungus to penetrate deeper into the susceptible cultivar. In pineapple fruitlets, the hyphae of *F. ananatum* mainly progressed directly between cell walls into the parenchyma but never reached the vascular region. A layer of thick-walled cells, in the case of the resistant cultivar, stopped the colonization, which were probably the infralocular septal nectaries. Anatomical and histochemical observations coupled with spectral analysis of the hypodermis suggested the role of lignin deposition in the resistance to *F. ananatum*. The major phenolics bound to the cell walls were coumaric and ferulic acids and were found in higher amounts in the resistant cultivar postinoculation. The combination of fruitlet anatomy and lignification plays a role in the mechanism of host resistance to fruitlet core rot.

## Introduction

Over the past decades, pineapple has become one of the leading commercial fruit crops worldwide. This development is the result of a globalization of pineapple production and the emergence of one particular cultivar. The breeding program led by the Pineapple Research Institute in Hawaii resulted in the creation of the cultivar “MD-2,” which is now the world’s leading pineapple (Bartholomew et al., 2012). This cultivar replaced the historical “Smooth Cayenne,” due to its higher yield and longer shelf life during shipment. Another cultivar, “Queen,” was not affected by these changes. The susceptibility of this pineapple to long periods of cold storage makes it mainly intended for the local fresh market. This cultivar is also susceptible to numerous pre- and postharvest diseases (Stewart et al., 2001; Luengwilai et al., 2016). However, consumers particularly appreciate its sweet and fruity taste and its exceptional aroma.

Damage caused by fruitlet core rot disease (FCR) depends on the pineapple cultivar. Although FCR is almost nonexistent in the “MD-2” cultivar, FCR is extremely worrisome for the “Queen” cultivar. In South Africa, losses due to FCR are far more serious than any other postharvest disease. The economic consequences led pineapple growers and researchers to investigate means of FCR control. Petty et al. (2005) sprayed a combination of two fungicides at flower induction and observed a significant reduction in the total number of black spots per fruit. Another program, aimed at controlling a vector mite, had the opposite effect to that expected: application of the miticide showed to increase the incidence of black spots (Manicom et al., 2006). However, the recent European Union restriction on the use of synthetic pesticides for their harmful effects on the environment and non-target organisms led the research to find alternatives ways to control pathogens. A better comprehension of the pathosystem is essential to consider effective treatments that are more respectful of the environment and living beings.

Morphological and anatomical structures influence host plant resistance to pathogens. Aquije et al. (2011) showed the structural differences between resistant and susceptible pineapple leaves to fusariose. The most recent descriptions of pineapple fruitlet core rot disease were of “Smooth Cayenne,” a moderately susceptible cultivar. The pathogen penetrates through the stigma during flowering, continues down the stylar canal into the locule, and frequently colonizes the placental tissue (Rohrbach and Apt, 1986). The fungus remains latent during fruit growth (Mourichon et al., 1987) and spreads once the fruits reach maturity. Fruitlet core rot has a complex etiology due to environmental conditions, fungal diversity, and pineapple physiology.

Few studies have characterized the biochemical changes that occur during pineapple fruitlet core rot disease. However, a large literature has described the physiological changes of the plant following a pathogen attack, and the phenylpropanoid pathway is often solicited (Dixon et al., 2002; Naoumkina et al., 2010; Miedes et al., 2014). Barral et al. (2017) showed an accumulation of free coumaroylisocitrate and caffeoylisocitrate in pineapple fruitlets following infection with *F. ananatum*. Hydroxycinnamic acids play an important role in plant–pathogen interactions with their antifungal properties and have an implication in lignin biosynthesis. Various biotic and abiotic stress conditions such as wounding, pathogen infection, and metabolic stress induce lignin biosynthesis, making cell walls rigid and impervious (Tronchet et al., 2010; Vanholme et al., 2010). Recent advances in spectral imaging suggest the role of lignin deposition in vanilla roots in the resistance to *F. oxysporum*. An algorithm connects the spectra of standard lignin compounds to the spectral image of the area of interest (Koyyappurath et al., 2015). Anatomical and histochemical observations coupled with biochemical analysis of cell wall-bound phenolics should lead to the development of novel approaches to enhance resistance durability.

In this study, we focused on host–pathogen interactions through host structures and cell wall composition. First, we described the morphological and anatomical differences of infructescence potentially related to FCR in resistant and susceptible pineapple cultivars. Second, we described the colonization pattern of *F. ananatum* in pineapple fruitlets and investigated the anatomy and kinetics of cell wall events associated with *F. ananatum* infection using the resistant “MD-2” and susceptible “Queen” pineapple cultivars. Finally, biochemical analyses of cell wall-bound phenolic compounds following inoculation were performed to confirm the spectral results.

## Materials and Methods

### Fungal Inoculum

The *F. ananatum* isolate Clp001 (CIRAD Collection, Ligne Paradis, Reunion Island) was used in this study. The fungal strain was isolated on naturally infected fruit on Reunion Island, purified, and integrated into the collection. This isolate exhibited high aggressiveness with repeatability when inoculated into the flesh of “Queen” and “MD-2” cultivars. For spore production, Clp001 isolate was grown on PDA plates and stored in the dark for 2 weeks at 25°C. The spores were recovered from the medium with sterile water, and the concentration of the conidial suspension was adjusted to 10^3^ micro and macroconidia per milliliter. Twenty-five microliters of the spore suspension was injected into the fruitlet using a 50-µL microsyringe (Hamilton Company, Reno, USA).

### Cell Wall-Bound Phenolic Monitoring

#### Plant Material

The experiment was conducted on a pineapple field of “MD-2” and “Queen” cultivars at the CIRAD Experimental Research Station (21°10´ South, 55°30´ East) in Reunion Island according to standard agricultural practices (Fournier, 2011). The inoculations were directly performed in November 2014 and January 2015 on mature green fruits in the field, 26 weeks after floral induction for “MD-2” and 19 weeks for “Queen” pineapples (80% of their expected harvest date). The analyses were carried out at 0, 2, 4, 6, 8, 10, 13, and 22 days postinoculation (dpi) for “Queen” and 0, 2, 4, 6, 8, 10, 14, and 25 dpi for “MD-2” cultivar. Four fruits were harvested at each sampling date for both cultivars. The level of maturity was determined according to the shell color. The letter G was assigned to green mature fruit, C2 as a half basal yellow fruit, and C4 as a totally yellow fruit (Darnaudery et al., 2016).

#### Phenolics Extraction and Identification

The infected fruitlets and healthy fruitlets were sampled separately for each fruit. The “infected fruitlets” modality corresponds to the fruitlets inoculated by *F. ananatum*, and the “healthy fruitlets” modality corresponds to fruitlets sampled on the opposite side of the fruit, as described by Barral et al. (2017). The fruitlets were dissected directly after harvest and dipped in liquid nitrogen. These frozen samples were ground in a Grindomix blender (Retsch, Haan, Germany), and the powders obtained were stored at −80°C. Frozen samples were lyophilized over 72 h at −52°C and a pressure of 0.3 mbar. Extraction of cell wall-bound phenolics was performed after several assays of the extraction solvent and mobile phase to optimize the saponification (Mertz et al., 2007; Mussatto et al., 2007; Tilay et al., 2008). One hundred milligrams of the dried powder was extracted twice for 10 min with 15 mL of 80% aqueous ethanol in an ultrasonic bath. Five milliliters of 2 N NaOH was added to the pellet for 2 h with stirring. The mixture was acidified to pH 2 with 3 N HCl and filtered with a 0.45-µm filter (Whatman plc, Maidstone, Kent, UK).

Identification was carried out on a UPLC-DAD-MS system. Separations were performed using a Waters Acquity UPLC-DAD system (Milford, MA, USA) on an Acquity BEH C18 column (150 × 1 mm i.d.; 1.7 μm; Waters, Milford, MA, USA), operating at 35°C. The mobile phase consisted of water/formic acid (99/1, v/v; eluant A) and methanol/formic acid (99/1, v/v; eluant B). The flow rate was 0.08 ml/min. The elution program was as follows: isocratic for 1 min with 2% B, 2–15% B (1–6.5 min), isocratic with 15% B (6.5–9 min), 15–30% B (9–12 min), isocratic with 30% B (12–14 min), 30–75% B (14–27 min), 75–95% B (27–32 min). ESI-MS/MS analyses were performed with a Bruker Daltonics Amazon (Bremen, Germany) mass spectrometer equipped with an electrospray source and an ion trap mass analyzer. The spectrometer was operated in positive and negative ion mode (end plate offset: −500 V; temperature: 200°C; nebulizer gas: 10 psi; dry gas: 5 l/min; capillary voltage: 2.5 kV in positive mode and 4.5 kV in negative mode). The collision energy for fragmentation used for MS^2^ experiments was set at 1 eV. Based on these identifications, changes in the levels of phenolic acids were monitored in healthy and infected pineapple fruitlets according to a previously published study (Barral et al., 2017).

### Anatomical Study of Pineapple Fruit

Fruit material consisted of commercially available pineapple fruit from Costa Rica for the “MD-2” cultivar and from the Reunion and Mauritius Islands for the “Queen” cultivar. The inoculated *in vitro* plants were examined for *F. ananatum* inoculation and colonization at 2, 4, and 6 dpi in at least two independent experiments. Control fruits were observed on the same dates as the inoculated fruits.

#### Wide-Field Microscopy

Fresh fruit sections (100 µm) obtained with a Microm HM650 V vibratome (Thermo Scientific, Walldorf, Germany) were dipped in a methyl blue (CI 42780) solution for 3 min to stain the fungi. Slides were observed with a Nikon Eclipse Ni-E (Tokyo, Japan) wide-field microscope (filter cube UV-2A, exc: 330-380, em: 420-800) with a PLAN APO 2x 0.1 NA objective and a Nikon CMOS DS-Ri2 camera, and images were processed with ImageJ v1.51n software.

X-Ray µ-Tomography:

A whole young fruit of the “Queen” cultivar was observed using a SkyScan 1076 microtomograph (Microphotonics, Belgium). The 3D reconstruction was performed with NRecon (Microphotonics, Belgium) and Avizo (FEI Visualization Sciences Group) software.

#### Scanning Electron Microscopy

Small pieces of fruit in the blossom cup region were observed with a Hitachi S4000 SEM.

#### Visualization of *F. ananatum* by Multiphoton Microscopy and Spectral Analysis

Fresh fruit sections (100 µm) obtained with the vibratome were mounted on a glass slide and observed with an LSM 880 multiphoton microscope (Zeiss, Jena, Germany) with a W-Plan-Apochromat 20x/1.0 objective equipped with a Chameleon Ultra II laser (Coherent, Santa Clara, CA, USA). Autofluorescence of the cell walls was observed at a wavelength of 720 nm, and detection was performed between 410 and 650 nm.

Image acquisition was performed using ZEN 2 software (Zeiss, Germany). The acquired images were merged and processed using ZEN 2 (Zeiss, Germany) and ImageJ v1.51n software.

#### Emission Spectral Analysis

The pulsed laser of the multiphoton microscope causes the excitation of secondary metabolites in a manner similar to that of a UV laser (Conéjéro et al., 2014; Talamond et al., 2015). Optimal excitation to observe the tissues of the fresh pineapple fruit was obtained at a wavelength λ = 720 nm with a bandpass emission in the 410- to 650-nm range using an array of 32 photomultiplier tube detectors (Zeiss), each with an 8.8-nm bandwidth. This spectral detector yielded spectral images and emission spectra in different parts of the fresh sections. The Linear Unmixing function was executed on these spectral acquisitions to separate, pixel by pixel, the mixed signals of four defined pure autofluorescent compounds: ferulic acid, sinapic acid, p-coumaric acid, and caffeoylquinic acid (Sigma-Aldrich, St. Quentin Fallavier, France), using the entire emission spectrum of each compound plus a residual channel. This image analysis showed each compound present in the sample with coded colors. In the residual channel, the intensity values represented the difference between the acquired spectral data and the fitted linear combination of the reference spectra.

All acquisitions were obtained using the facilities of the regional MRI (Montpellier Ressources Imagerie) platform, a member of France BioImaging.

## Statistical Analysis

All statistical analyses were conducted in R (R Development Core Team 2015). A Box-Cox transformation was performed to ensure normal distributions of residues and the homogeneity of variance of residuals. Analyses of variance (ANOVA) on phenolic acid levels between sampling dates were carried out. Comparisons of the means in terms of the phenolic acids among the fruitlets were statistically evaluated for each sampling date using Tukey’s multiple comparison tests.

## Results

### Pineapple Fruitlet Overview

**Figure 1:**
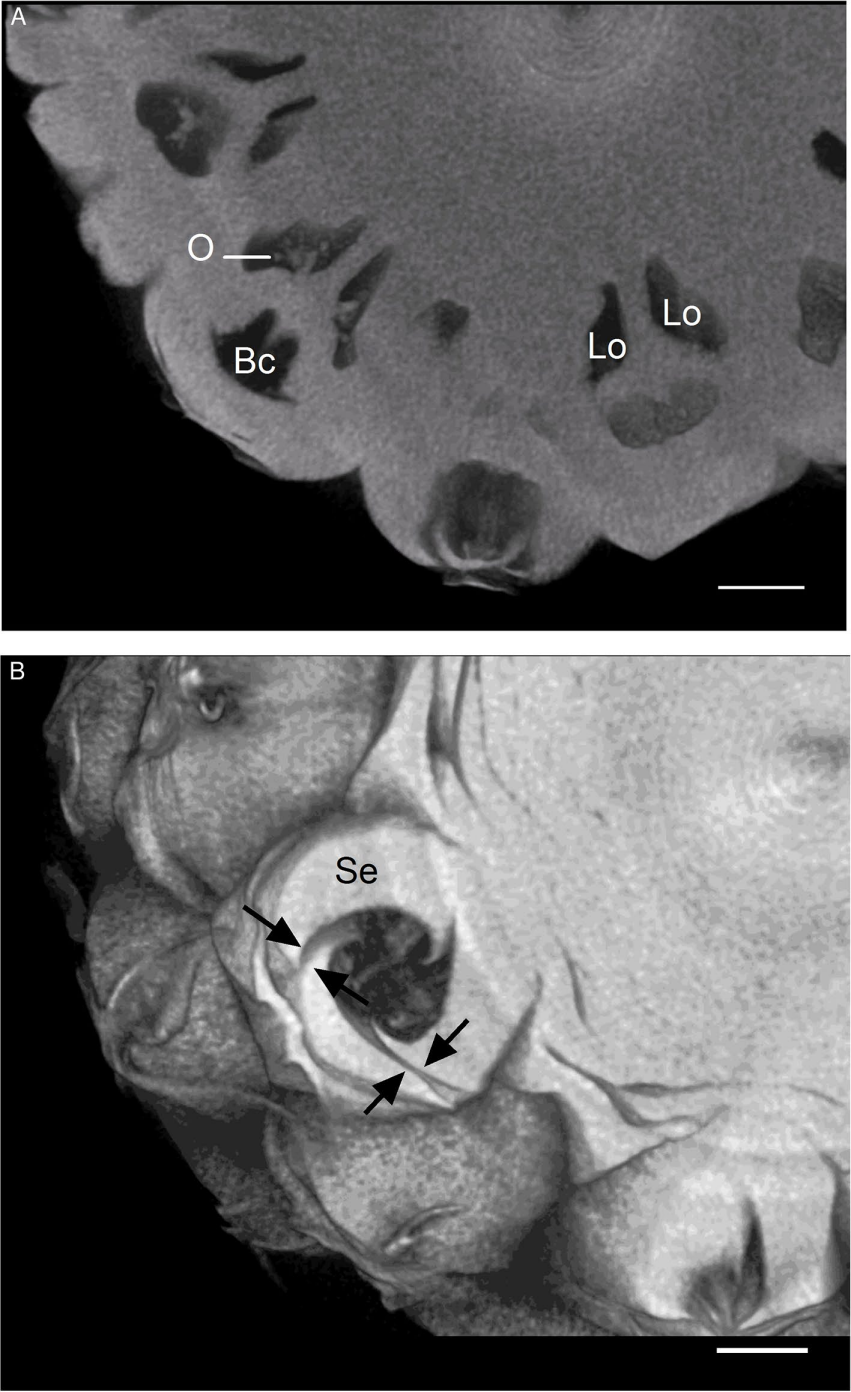
3D reconstructed dataset visualizing transverse sections of pineapple fruit. **(A)** Fruitlets. **(B)** Blossom cup; black arrows indicate imperfect fusions between sepals. Bc, blossom cup; Lo, locule; O, ovule; Se, sepal (scale bar = 1 cm).

Transverse sections of the pineapple fruit of the “Queen” cultivar were observed with an X-ray microtomograph. **Figure 1** shows syncarpic infructescence resulting from the fusion of the basal part of the flowers and their ovaries, separated by the parenchymatous tissue of the sepals and bract bases. The blossom cup corresponds to a floral cavity that is surrounded by bracts and sepals fused with each other at their base (**Figure 1A**). The apical part of the sepals did not fuse with each other, and the bracts did not unite with the sepals (**Figure 1B**). Spaces between two sepals up to 100 µm were observed (arrows). Mites and ants were frequently found in the floral cavities of the mature fruit during the trial. The withered style is visible in the middle of the cavity and is inserted at the base of the ovary. The ovary below the blossom cup is tricarpellate and trilocular, with the three septa forming an inverted Y when seen in the tangential section of the inflorescence. The ovules are located in the upper part of the deep cavities, known as locules (Coppens d’Eeckenbrugge and Leal, 2003).

### Blossom Cup

**Figure 2:**
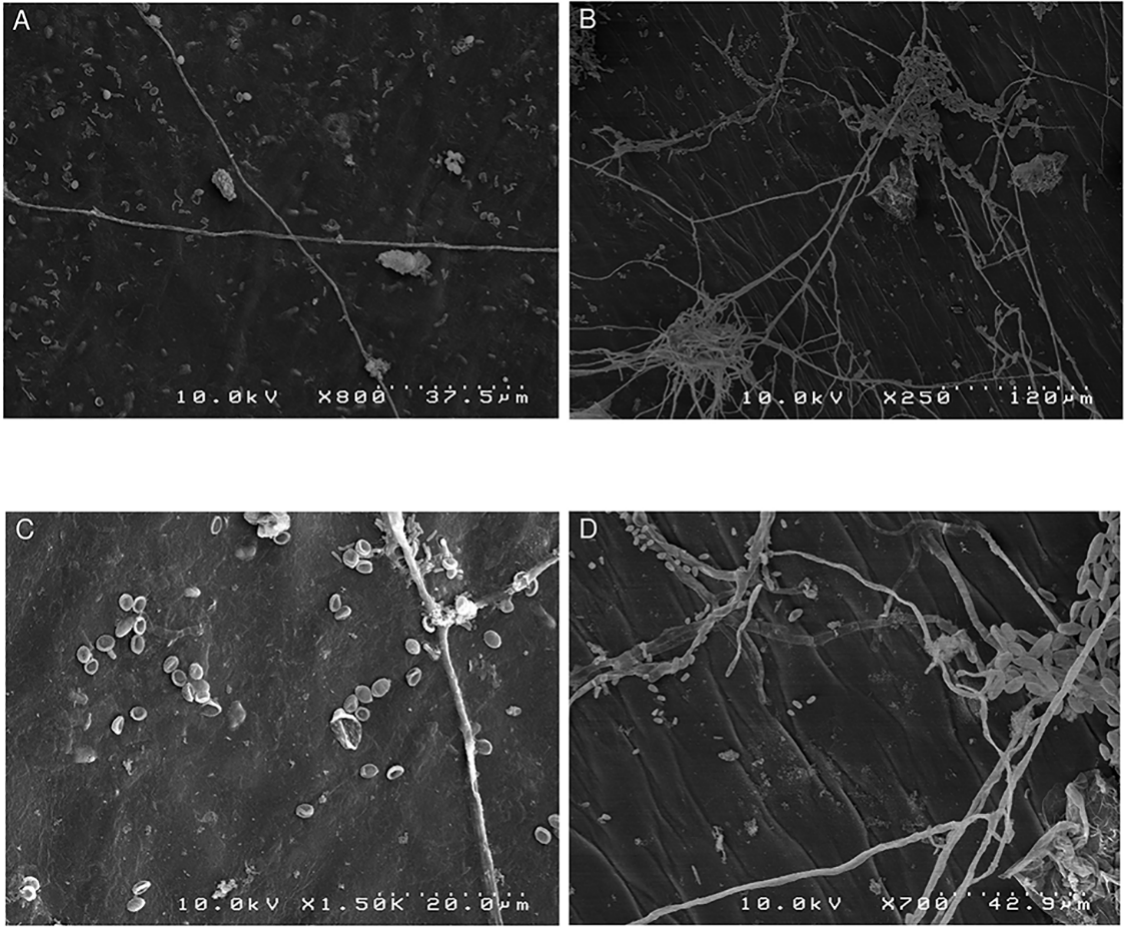
Scanning electron microscopy images of natural fungal colonization of the asymptomatic blossom cup of “MD2” and “Queen,” which are, respectively, the resistant and susceptible cultivar to pineapple fruitlet core rot disease. **(A)** Represents the sparse mycelial network of the “MD-2” blossom cup and **(B)** the dense mycelial network in “Queen.” **(C)** Spores with ellipsoidal shapes dispersed in the “MD-2” cultivar. **(D)** Displays spores with a fusiform shape in a “Queen” blossom cup.

A scanning electron microscope examination of the blossom cup indicated the presence of fungi in both cultivars under natural conditions (**Figure 2**). The surface of the “MD-2” floral cavity was partially covered by mycelia and spores with an ellipsoidal shape (**Figures 2A, C**). The micrographs of a symptomless “Queen” blossom cup showed a dense mycelial network with fusiform spores characteristic of *F. ananatum* (Jacobs et al., 2010) (**Figures 2B, D**). The microorganisms were isolated from the floral cavity and grown in pure culture to which chloramphenicol was added. The pathogen was found in all floral cavities regardless of the cultivar.

**Figure 3:**
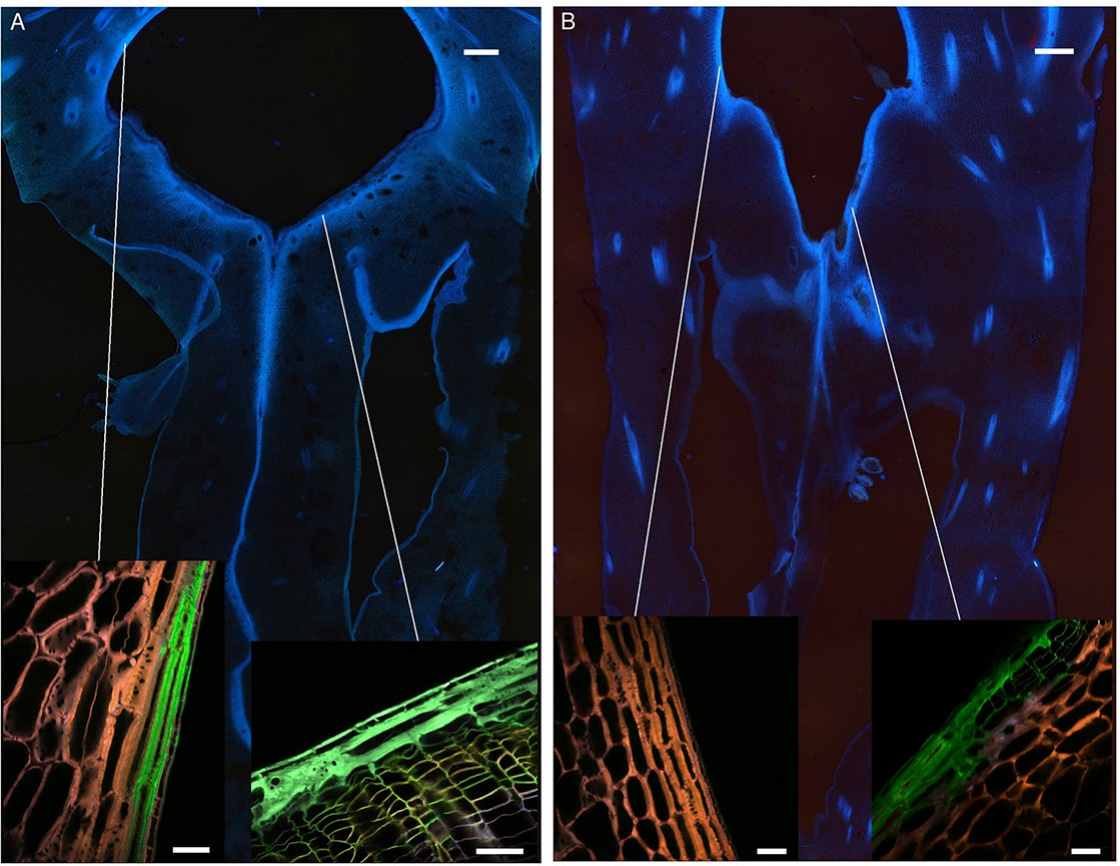
Fruitlet cross sections observed with an epifluorescence microscope of the **(A)** “MD-2” resistant pineapple cultivar and **(B)** “Queen”, susceptible cultivar (filter exc: 340–380 nm, em: 425–800 nm) (scale bar = 1,000 µm). The close-up rendering illustrates spectral-merged images of the lining cell wall composition of the blossom cup observed with a multiphoton microscope, using four reference emission spectra compounds: ferulic acid = green color; p-coumaric acid = blue color; sinapic acid = yellow color; and caffeoylquinic acid = red color (scale bar = 50 µm).

Transversal sections were carried out on uninfected fruitlets of both cultivars. The shape of the blossom cup is more flattened and flared in the “MD-2” cultivar (**Figure 3A**) than in the “Queen” (**Figure 3B**). The lining of the cavity differs in thickness, composition, and continuity between the two cultivars. Spectral analyses of the epidermal layers of the floral cavity of the susceptible “Queen” and resistant “MD-2” cultivar were compared with the reference spectra of the lignin precursors (see close-ups in **Figure 3**). Based on the fluorescence of these phenolic compounds, the images are similar for both cultivars in the style and stamen regions. Ferulic acid is the major compound of the cell walls, followed by *p*-coumaric acid. The “MD-2” cell walls of the outer layer retain the same composition, even in the regions the furthest away from these floral parts. On the other hand, images of the “Queen” pineapple demonstrate a loss of the characteristic fluorescence signal of the cell wall phenolics. In the susceptible cultivar, the outer layer colored with ferulic acid is discontinuous or very thin, while the resistant cultivar shows a strong and continuous lignin thickening on the outer walls of the blossom cup.

### Carpel Delimitation

**Figure 4:**
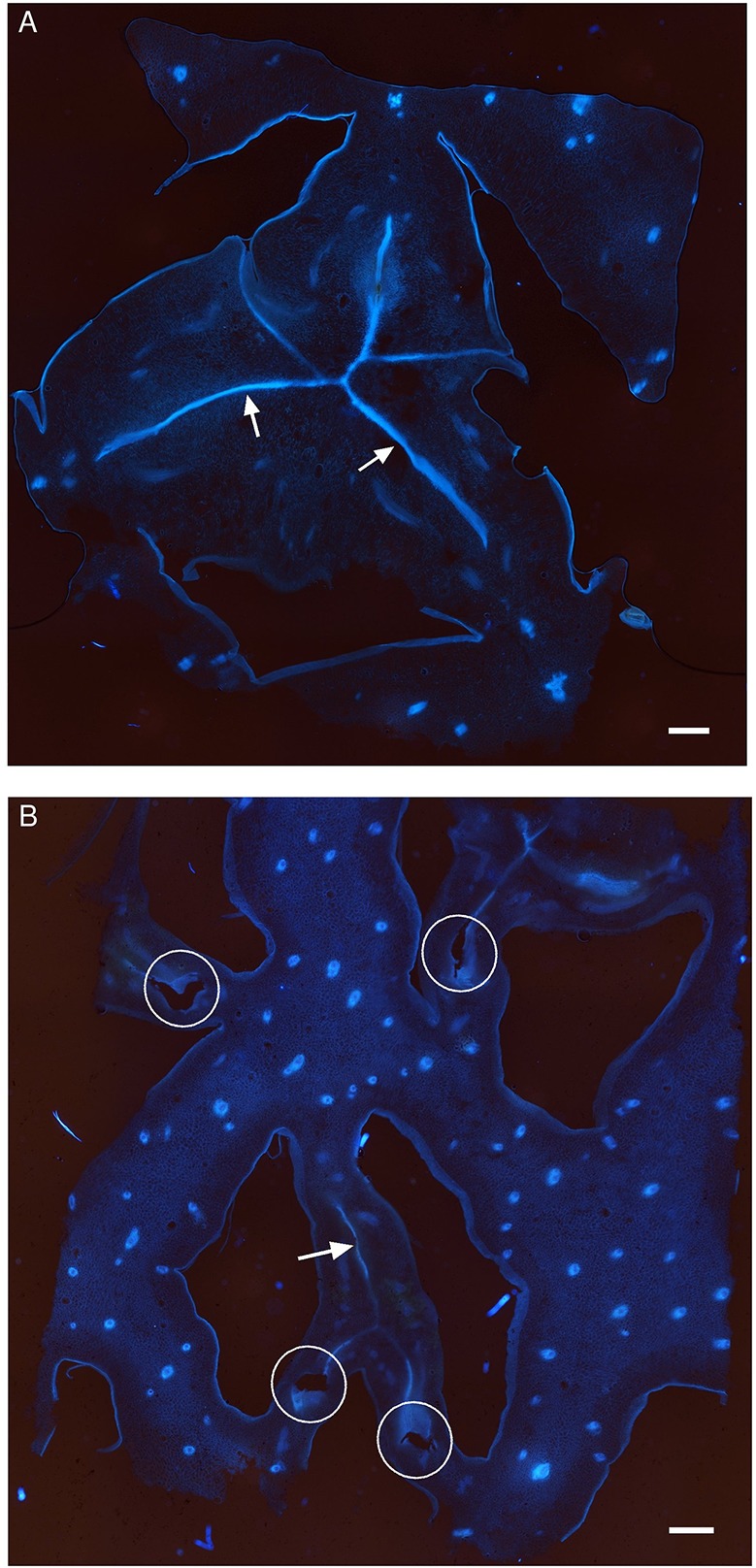
Autofluorescence images of the ovary in a cross section of pineapple fruits observed with an epifluorescence microscope (filter exc: 340–380 nm, em: 425–800 nm) in the **(A)** “MD-2” resistant cultivar and **(B)** “Queen” susceptible cultivar; white arrows indicate carpel margin; white ellipses indicate septal nectaries; Lo, locule.

Cross-sections of the ovaries at the placenta level were observed under UV excitation to visualize a blue autofluorescence of the cell walls (**Figure 4**). The septa separating the three carpels are less conspicuous in the “Queen” cultivar than in “MD-2” (arrows). The line where the carpels are facing each other is thick and continuous in the “MD-2” ovary (**Figure 4A**), unlike “Queen” where the fluorescence signal is weak and discontinuous (**Figure 4B**). The empty spaces indicated by the ellipses correspond to septal nectaries. This anatomical feature is present only in the “Queen” cultivar and not even for all carpel margins.

### Colonization of the Parenchyma

**Figure 5:**
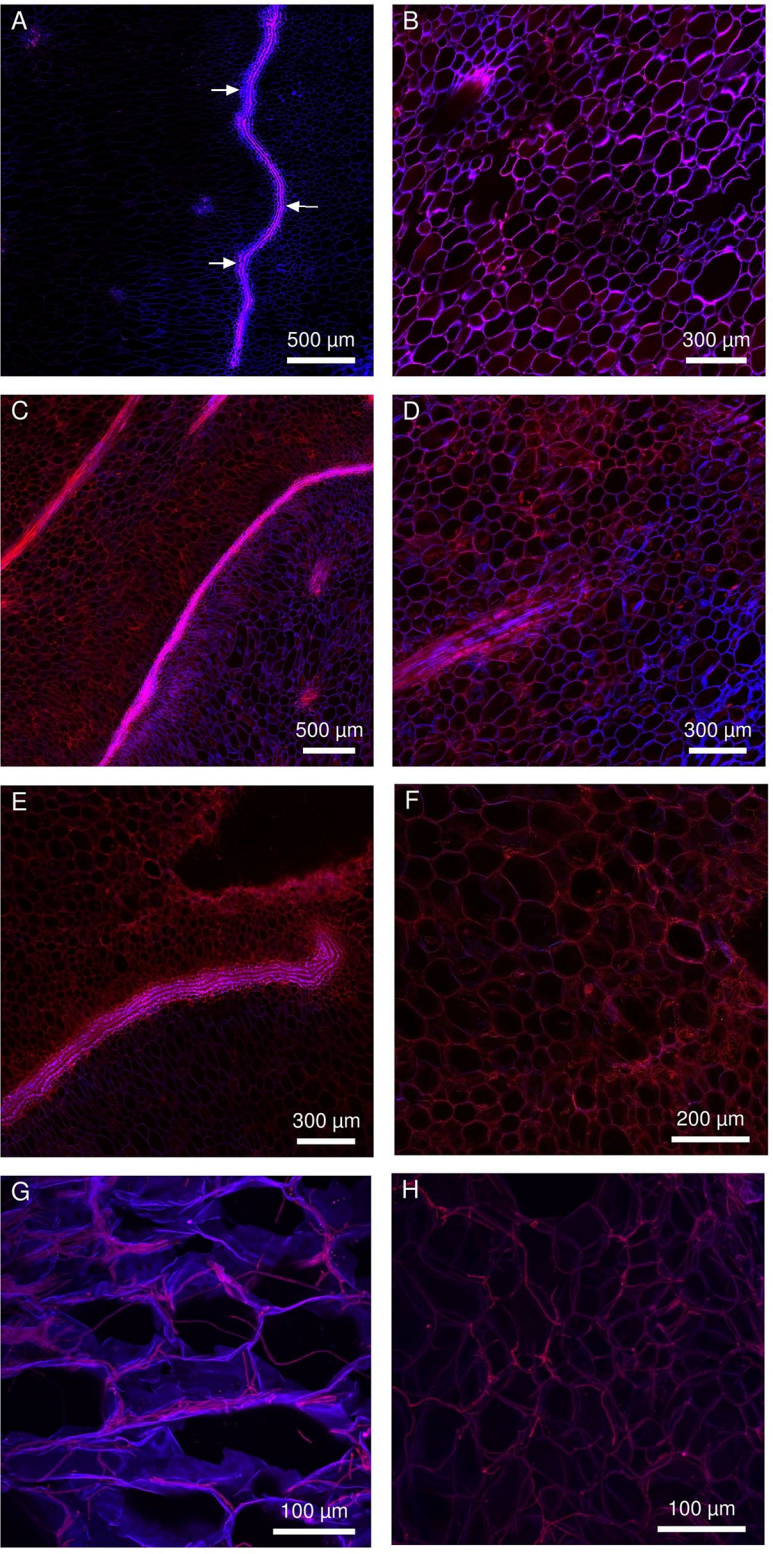
Multiphoton microscopic images showing the colonization pattern of *Fusarium ananatum* stained with methyl blue in the parenchyma of the resistant fruitlet pineapple cultivar “MD-2” (left column) and the susceptible cultivar “Queen” (right column). **(A, B)** Interparietal hyphae progression at 2 days postinoculation. **(C, D)** At 4 days postinoculation, the thick-walled cell barrier blocked hyphae progression in “MD-2” **(C)**, while hyphae continued to develop in “Queen” parenchyma **(D)**. **(E, F)** At 6 dpi, hyphae bypassed the lining limit in “MD-2” **(E)** and continued to develop in “Queen” parenchyma **(F)**. **(G, H)** 3D reconstructed Z-stack images of hyphae progression in pineapple fruit parenchyma at 5 days postinoculation (exc: laser IR 720 nm, em: channel 435–485 nm in blue, channel 670–700 nm in red).

*Fusarium ananatum* was inoculated just below the blossom cup in both cultivars, and the progression of the hyphae was monitored in the parenchyma (**Figure 5**). The fungus was detected in the parenchyma of both genotypes from the second day after inoculation. A labyrinthine layer of thick-walled cells passed through the parenchyma of the resistant cultivar (**Figure 5A**, arrows). This particular constitutive structure of “MD-2” was not observed in the susceptible “Queen” pineapple (**Figure 5B**). The blue and red emission channels revealed cell walls and hyphae, respectively. At 2 days postinoculation, the hyphae had already spread into the parenchyma of the susceptible and resistant pineapple fruitlets. Inoculations resulted in fungal colonization and the death of tissue in the infected area, characterized by a decrease in signal. At 4 days postinoculation, the layer of thick-walled cells blocked the fungal colonization in “MD-2” (**Figure 5C**). In contrast, hyphae were present in large areas in the “Queen” cultivar, with no structure to slow them down (**Figure 5D**). At 6 days postinoculation, the hyphae bypassed the “MD-2” barrier (**Figure 5E**) and largely occupied the “Queen” parenchyma (**Figure 5F**), with the bright red color emphasizing the pathogen density. The layer of thick-walled cells slowed the progression of the hyphae without completely stopping it. Phloem and xylem were not affected by the pathogen even 6 days postinoculation (data not shown). The fungi preferentially colonized the tissues by apoplasm but were able to penetrate and spread through the cells (**Figures 5G, H**).

### Subsequent Biochemical Composition Changes of the Labyrinthine Layer in the Resistant Cultivar

**Figure 6:**
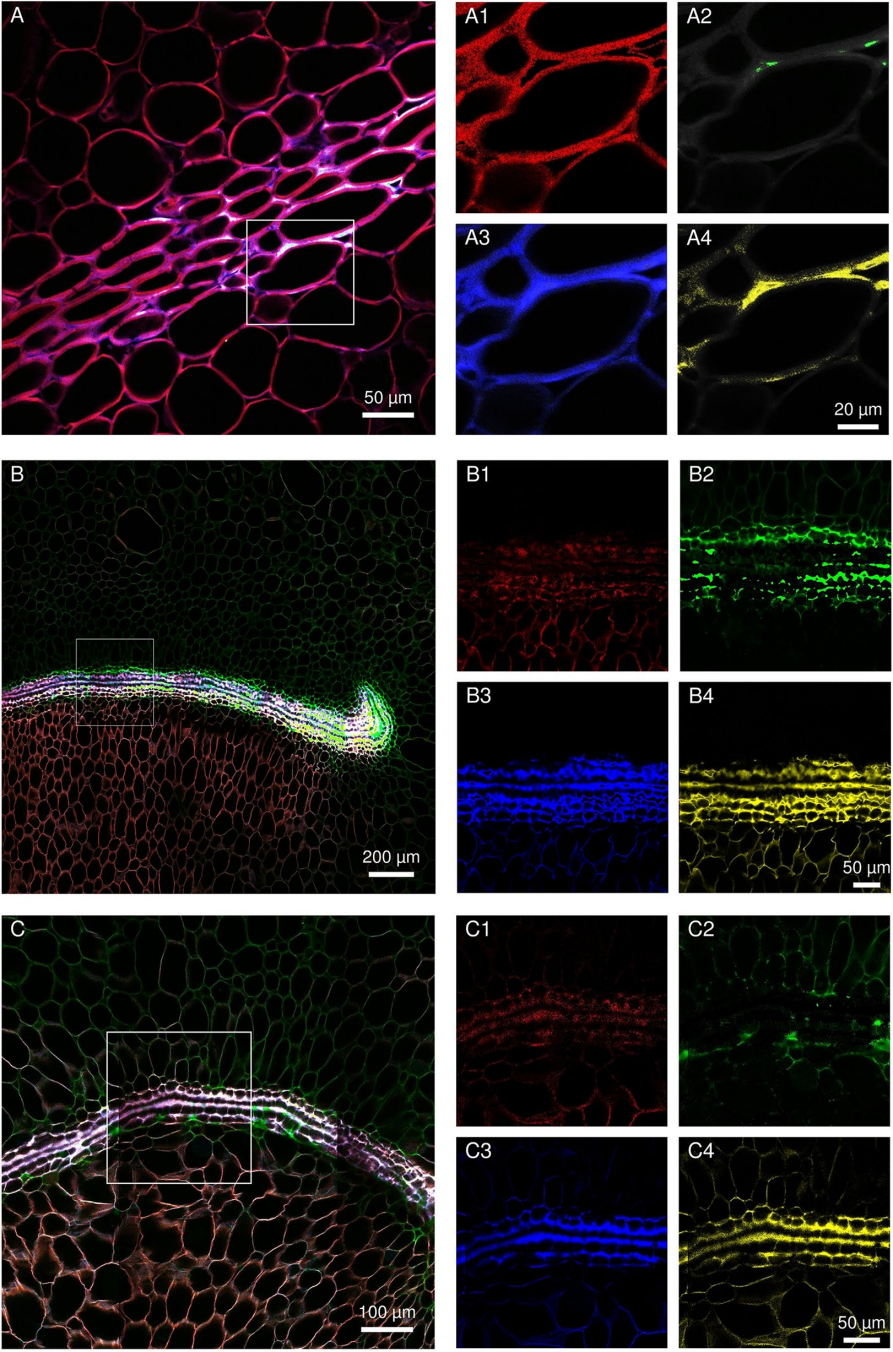
Evolution of septal nectary cell wall histochemical composition following *Fusarium ananatum* inoculation in the resistant “MD-2” pineapple. **(A)** Spectral image of septal nectary before inoculation. **(B, C)** Histochemical composition of septa nectaries at different levels 2 days postinoculation. Merged images **(A**–**C)** were split into four base images (1, 2, 3, and 4). The close-up (1) illustrates histolocalization of caffeoylquinic acid; (2) ferulic acid; (3) p-coumaric acid; and (4) sinapic acid.

Spectral analyses of the infected regions of susceptible and resistant cultivars were compared with reference spectra of four lignin precursors and constituents (chlorogenic acid, ferulic acid, sinapic acid, and *p*-coumaric acid). **Figure 6** shows the evolution of the cell wall histochemical composition of the labyrinthine layer in “MD-2” following *F. ananatum* inoculation. This thick layer and parenchyma cell walls had a similar composition before inoculation (**Figure 6A**). After inoculation, the layer of thick-walled cells displayed very divergent unmixed spectra compared to preinoculation. Ferulic and *p*-coumaric acids were the main constituents binding the thick cell wall layer after fungal inoculation (**Figures 6B, C**). The cell wall composition in the parenchyma also evolved according to fungal colonization. The upper part of the image shows the healthy parenchyma and the lower part shows the infected parenchyma. The cell walls exhibited a green color characteristic of ferulic acid in the upper part and a brown color in the lower part 2 days postinoculation.

### Increase in Cell Wall-Bound Phenolics After *F. ananatum* Infection

**Figure 7:**
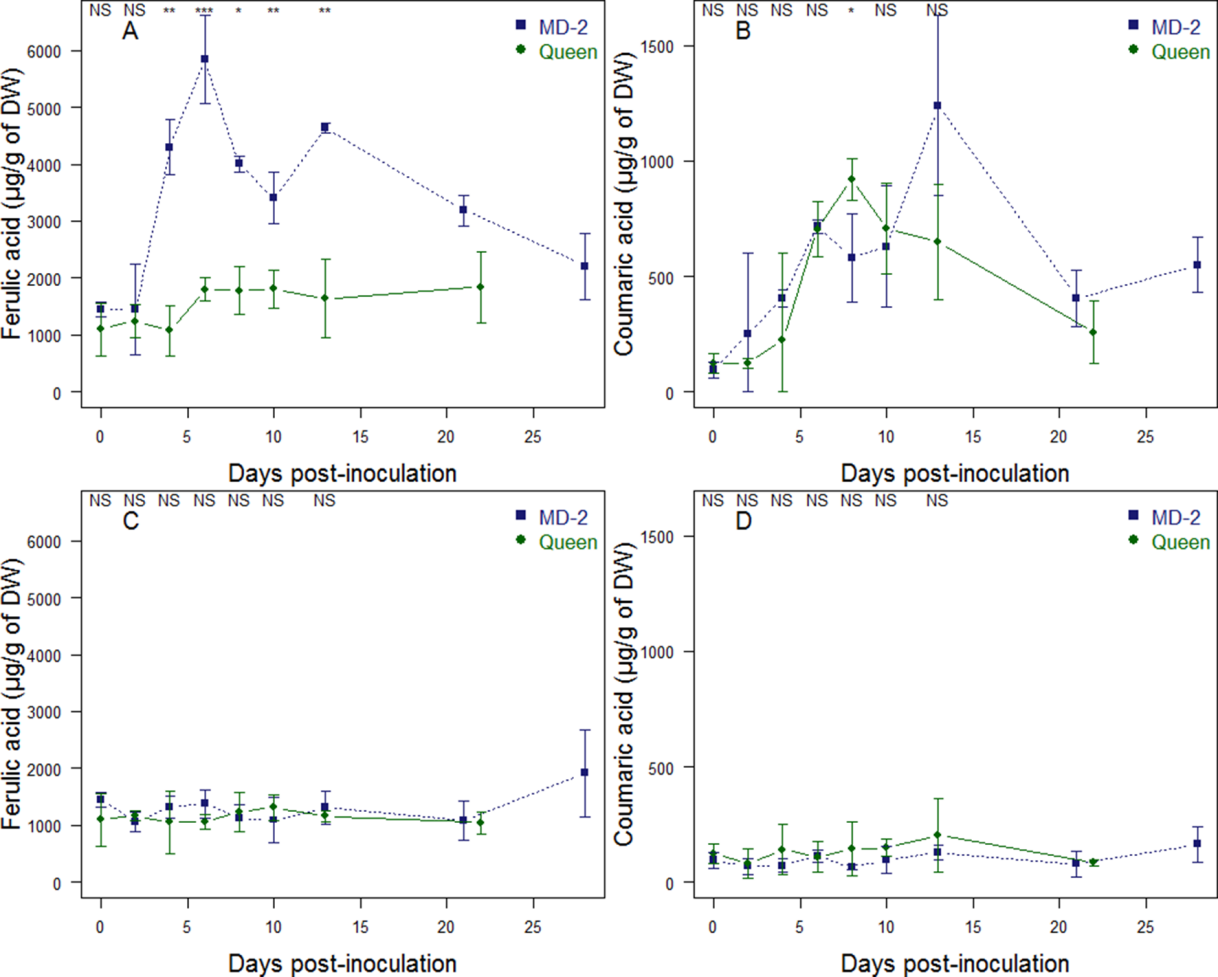
Evolution of cell wall-bound ferulic acid **(A, C)** and *p-coumaric* acid **(B, D)** in “Queen” (•) and “MD-2” (▪) pineapple fruits; in infected fruitlets after *Fusarium ananatum* inoculation **(A, B)**; and in healthy fruits during natural ripening **(C, D)**. Vertical bars represent standard error of means (n = 4 for healthy fruitlets and n = 3 for infected fruitlets). Differences between cultivars were either significant at P < 0.05 (∗), P < 0.01 (∗∗), P < 0.001 (∗∗∗), or nonsignificant (NS) for each sampling date.

The evolution of cell wall-bound phenolics in “MD-2” and “Queen” pineapple fruits was monitored in pineapple fruitlets after *F. ananatum* inoculation and during natural ripening (**Figure 7**). UPLC-MS made it possible to identify *p*-coumaric acid and ferulic acid as the only phenolic compounds of the pineapple fruit cell walls. Hydroxycinnamic acids are found in both healthy and infected fruitlets with varying patterns of evolution. In the infected fruitlets of the resistant cultivar, the level of ferulic acid significantly increased 2 days after inoculation, reaching a maximum of 5,841 µg g^−1^ of dry weight (DW). In comparison, the ferulic acid level significantly increased 6 days postinoculation, reaching 1,798 µg g^−1^ of DW in the susceptible cultivar and then remained stable until the end of the experiment. (**Figure 7A**). Concerning *p*-coumaric acid, fungal inoculation of *F. ananatum* generated a direct response in the resistant cultivar. In the susceptible cultivar, changes were detectable within 4 days postinoculation (**Figure 7B**). The *p*-coumaric acid level reached a maximum at 13 dpi with 1,239 µg g^−1^ of DW for “MD-2.” In “Queen” infected fruitlets, the peak was 920 µg g^−1^ of DW at 8 dpi. The resistant “MD- 2” pineapple had a shorter time and higher level of response to fruitlet core rot infection than the susceptible “Queen” pineapple. After those respective maximums were reached, the level of cell wall-bound phenolics slightly decreased to a level similar to that found in the healthy fruitlet.

The evolution of cell wall-bound phenolics in healthy fruitlets was monitored according to maturity stages (**Figures 7C, D**). The ferulic acid level is not significantly different between “MD-2” and “Queen” cultivars and remains stable throughout fruit maturation, except at the last stage of ripening of “MD-2,” where the level of ferulic acid significantly increases from 1,086 µg g^−1^ to 1,915 µg g^−1^. The coumaric acid level is approximately 10 times lower than the level of ferulic acid in healthy fruitlets. “MD-2” and “Queen” healthy fruitlets are not significantly different in terms of coumaric acid level.

## Discussion

Fruitlet core rot has affected the pineapple industry for decades. Much research was conducted to eradicate the pathogen at the assumed moment of its penetration (Rohrbach and Pfeiffer, 1976; Mourichon, 1983). The experiments gave mixed results, partly because of a lack of knowledge of the host–pathogen interaction. In this study, complementary tools such as spectral cell imaging linked to accurate biochemical analyses have increased knowledge about host–pathogen interactions. First, the anatomy of the fruitlet was precisely described, and several anatomical structures involved in FCR resistance were unveiled. Second, the colonization pattern of *F. ananatum* in the resistant and susceptible cultivars revealed the predominant role of septal nectaries on colonization inhibition. Finally, biochemical analyses confirmed the involvement of cell wall-bound phenolics in the resistance to fruitlet core rot previously highlighted using image acquisition.

All of the previous studies concurred that the fungus penetrates at the time of flowering. The pathogen was never observed on our cross-sections of the flowers. Tadych et al. (2012) showed on the cranberry fruit rot disease that certain species of fungi are absent at flowering but present at the maturity of the fruit and vice versa. In addition, the same species colonize both susceptible and resistant varieties. To check if the fungi are present at the time of flowering, it would be interesting to isolate the different floral parts at the time of flowering on growth medium. However, the presence of mealybugs and other insects in the floral cavity of the mature fruit was observed in the “Queen” cultivar (personal observation). There is therefore a postflowering means to access the floral cavity. In the literature, the floral cavity is described as a fusion of the bracts and sepals (Okimoto, 1948). A 3D X-ray tomography showed that this fusion was imperfect, leaving sufficient space for fungi and other insects to get through. To confirm these observations, the causal agent was found growing on floral remnants of the blossom cup. Hyphae and characteristic spores of *F. ananatum* covered the surface of the susceptible cultivar, whereas the hyphal network was more scattered on the resistant cultivar. Fungi were found in the blossom cups of both cultivars, but the symptoms of the disease were expressed only in the susceptible cultivar. Other mechanisms are therefore involved in disease resistance.

Spectral analysis using a multiphoton microscope made it possible to differentiate the lining of the blossom cup. The cell walls of the outer layer are mainly composed of ferulic and coumaric acids. In the susceptible “Queen” blossom cup, the presence of lignin is discontinuous or very thin, whereas the resistant “MD-2” blossom cup shows a strong and continuous lignin thickening on the outer cell walls. Lignin is known to be a physical barrier to pathogen progression (Nicholson and Hammerschmidt, 1992). It is assumed that lignified cell walls hamper hyphal penetration and colonization of the intercellular spaces. Moreover, the lining of the “Queen” blossom cup is occasionally cracked, with numerous mycelia observed around it. Oxenham (1953) mentioned the possibility that the pathogen enters the pineapple flesh from growth cracks or insect feeding areas. Ripening causes the disintegration of the walls and occasional cellular breakdown in pineapple fruitlets (Okimoto, 1948). The pathogen could take advantage of these weaknesses to penetrate the flesh.

The transverse section of the ovary revealed a cellular layer clearly delimiting the carpels in the resistant cultivar. However, the susceptible cultivar exhibited a low-intensity fluorescence with discontinuities in the layer characteristic of necrotic tissues. During carpel development, the sides where two carpels meet sometimes fail to close up completely and leave a small, narrow, elliptical opening extending downward between the carpels, from the bottom of the blossom cavity of the cup, permitting air to enter these spaces (Kerns et al., 1936). As the fruit develops to maturity, the cells of the carpel walls are thus exposed to air and become necrotic tissue by oxidation. Only the “Queen” cultivar has this anatomical feature in our observations.

Pineapple is a monocotyledonous plant that possesses trimeric flowers with three sepals, three petals, and three carpels. In the Bromeliaceae family, to which pineapple belongs, carpels are presented in an axillary position. The ovary consists of three locules delimited by three carpels facing each other, as seen on the cross-sections of the resistant cultivar (carpel margin). Sajo et al. (2004) showed the presence of interlocular nectaries in some Bromelioideae that open to the hypanthium floor. They form at the unfused regions of otherwise fused carpel margins. The nectaries are glandular tissues secreting mainly sugars offered to pollinators as a reward (Stiles and Freeman, 1993; Proctor et al., 1996). The pathogen takes advantage of this duct to penetrate deeper into the fruit of the susceptible cultivar. Moreover, the early symptoms of FCR often are found in this area.

The progression of the pathogen was monitored in order to understand what may slow down or even block its development in the parenchyma. Since the symptoms are not external, *F. ananatum* was inoculated directly into the flesh to track its progression over time in both cultivars. At 2 days postinoculation, both cultivars exhibited fungal growth in the flesh of the fruit. The hyphae invaded the parenchyma mainly through the intercellular spaces, but tips of hyphae were observed in cells. These unlignified cell walls were histochemically shown to be noncellulosic polysaccharides (Smith and Harris, 1995). The pathogenic fungi easily progress in this kind of interparietal structure. At 4 days postinoculation, the fungus spread continuously into the parenchyma of the “Queen” cultivar.

In the resistant cultivar, a labyrinthine layer of thick-walled cells blocked hyphae development. This anatomical feature is very similar to what is described as an infralocular nectary by Sajo et al. (2004) and Rudall (2002). This constitutive layer prevents fungus from spreading any further into the flesh. Spectral analysis showed differences in cell wall composition between parenchyma and septal nectaries after infection. Ferulic and coumaric acids are the main constituents of these lignified cell walls. The structural complexity of lignin, its high molecular weight, and its insolubility make its degradation very difficult (Pérez et al., 2002). Currently, the ability of *F. ananatum* and *Talaromyces stollii* to degrade lignin is unknown. This layer, with large amounts of ferulic and coumaric acids only in the resistant genotypes, suggests the important role of lignified walls. Accumulation of ferulic acid occurs in the parenchyma near the infected area. Parenchyma cells are unlignified but contain ester-linked ferulic acid (Smith and Harris, 2001). This increase in phenolic acids may be a preventive defense mechanism for fungal colonization.

*Fusarium ananatum* never reached the parenchyma vascular system even at 6 days postinoculation, indicating that the spread of the fungus was mainly due to the destruction of the parenchyma cells. Phloem and xylem were not affected by the pathogen, which is perhaps the reason why the disease does not spread all over the fruit and remains restricted to small areas. The members of the *Fusarium* genus are generally vascular and cause sudden wilt in plants by rapidly invading the vascular bundles (Ndambi et al., 2012).

Based on these observations, the composition of cell walls seems determinant in the resistance to fruitlet core rot. We therefore biochemically monitored the evolution of cell wall-bound phenolics during natural ripening and postinoculation with *F. ananatum*. In reaction to the infection, plant-induced resistance leads to an accumulation of ferulic acid and, to a lesser extent, of coumaric acid. Besides being a key component of lignin, ferulic acid crosslinks with polysaccharides upon attack by a pathogen, increasing the cell wall resistance to digestion by microbial cell wall-degrading enzymes (Bily et al., 2003; Passardi et al., 2004; Bellincampi et al., 2014). The resistant cultivar exhibits a faster and stronger response to fungal inoculation in terms of accumulation of cell wall-bound phenolics in the infected fruitlets compared to the susceptible cultivar. This factor could also be responsible for the higher resistance of “MD-2” cultivar to fungal colonization than “Queen.” In a previous study, we found large amounts of coumaroylquinic and hydroxybenzoic acids as soluble phenolic compounds in the flesh of healthy mature fruitlets of “MD-2” (Barral et al., 2019). Their availability may explain the rapid accumulation of ferulic acid in the cell walls of the resistant variety. Furthermore, the amount of ferulic acid significantly increased in the resistant cultivar during the late stages of natural ripening. However, the susceptible cultivar exhibited no change in the amount of ferulic acid. Moreover, the symptoms of FCR appear naturally at this stage of maturity in the “Queen” fruits. Phenolic polymers have a direct effect on fungi as a structural barrier, but free phenolic compounds such as ferulic and coumaric acids have antimicrobial activities (Daglia, 2012; Barral et al., 2017). This change in hydroxycinnamic acids may contribute to slowing down the fungal pathogen.

Altogether, our analyses revealed a considerable difference in the physical properties of the resistant and susceptible cultivars, with more structural integrity associated with higher levels of cell wall-bound phenolics found in the resistant cultivar.

## Author Contributions

BB, MC, MLa, GC and SS-G contributed to the conception of the work; to the acquisition and analysis of data. BB, MLé and J-LV helped for interpretation and revised the manuscript.

## Conflict of Interest Statement

The authors declare that the research was conducted in the absence of any commercial or financial relationships that could be construed as a potential conflict of interest.
